# Usefulness and applicability of the revised dengue case classification by disease: multi-centre study in 18 countries

**DOI:** 10.1186/1471-2334-11-106

**Published:** 2011-04-21

**Authors:** Judit Barniol, Roger Gaczkowski, Eliana Vega Barbato, Rivaldo V da Cunha, Doris Salgado, Eric Martínez, Carmita Soria Segarra, Ernesto B Pleites Sandoval, Ajay Mishra, Ida Safitri Laksono, Lucy CS Lum, José G Martínez, Andrea Núnez, Angel Balsameda, Ivan Allende, Gladys Ramírez, Efren Dimaano, Kay Thomacheck, Naeema A Akbar, Eng E Ooi, Elci Villegas, Tran T Hien, Jeremy Farrar, Olaf Horstick, Axel Kroeger, Thomas Jaenisch

**Affiliations:** 1 Section Clinical Tropical Medicine, Department for Infectious Diseases, University Hospital Heidelberg, Im Neuenheimer Feld 324, 69120 Heidelberg, Germany; 2 Hospital Municipal Francés, Santa Cruz de la Sierra, Santa Cruz, Bolivia; 3 Universidad Federal de Mato Grosso do Sul, Brazil; 4 Hospital Universitario de Neira, Universidad Surcolombiana, Neira, Colombia; 5 Instituto de Medicina Tropical Pedro Kouri, Autopista Novia del Mediodía Km6, PO Box 601, Marianao 13, Ciudad de la Habana, Cuba; 6 Hospital de Infecciología, Guayaquil, Ecuador; 7 Hospital Nacional de Niños Benjamin Bloom, Final 25 Avenida Norte y Boulevar de los Héroes, San Salvador, El Salvador; 8 Sunderlal Memorial Hospital, Dilshad Garden, Delhi 110095, India; 9 Tropical and Infectious Disease Sub Division, Paediatric Department, Dr. Sardjito Hospital/Faculty of Medicine, Gadjah Mada University, Yogyakarta, Indonesia; 10 Department of Paediatrics, Faculty of Medicine, University of Malaya, Kuala Lumpur, Malaysia; 11 Servicios de Salud de Nuevo León, Monterrey, Nuevo León, México; 12 Centro Nacional de Diagnóstico y Referencia, Ministerio de Salud, Managua, Nicaragua; 13 Dirección General de Vigilancia de la Salud/Estrategia de Gestión Integrada para prevención y control del Dengue en Paraguay, Asunción, Paraguay; 14 Dirección de Salud II Lima Sur, Ministerio de Salud, Jr. Martínez de Pinillos 124-B, Lima 4, Perú; 15 San Lazaro Hospital, San Lazaro Compound, Quiricada Street, Sta Cruz, Manila 1003, Philippines; 16 Centers for Disease Control and Prevention, Division of Vector Borne Infectious Diseases, 1324 Calle Cañada, San Juan, Puerto Rico; 17 Preventive Affair Department, Jeddah Governorate, MOH, P.O. Box 54165 Jeddah 21514, Saudi Arabia; 18 Duke-NUS Graduate Medical School, 8 College Drive, Singapore 169857; 19 Instituto Experimental José Torrealba, Núcleo Universitario Rafael Rangel, Universidad de los Andes, Trujillo, Venezuela; 20 Hospital for Tropical Diseases 190 Ben Ham Tu, Quan 5 Ho Chi Minh City, Viet Nam; 21 Oxford University Clinical Research Unit, Hospital for Tropical Diseases, Ho Chi Minh City, Viet Nam; 22 Special Programme for Research and Training in Tropical Diseases, World Health Organization, Avenue Appia 20, CH-1211 Geneva 27, Switzerland; 23 Liverpool School of Tropical Medicine, Pembroke Place, Liverpool, UK

## Abstract

**Background:**

In view of the long term discussion on the appropriateness of the dengue classification into dengue fever (DF), dengue haemorrhagic fever (DHF) and dengue shock syndrome (DSS), the World Health Organization (WHO) has outlined in its new global dengue guidelines a revised classification into levels of severity: dengue fever with an intermediary group of "dengue fever with warning sings", and severe dengue. The objective of this paper was to compare the two classification systems regarding applicability in clinical practice and surveillance, as well as user-friendliness and acceptance by health staff.

**Methods:**

A mix of quantitative (prospective and retrospective review of medical charts by expert reviewers, formal staff interviews), semi-quantitative (open questions in staff interviews) and qualitative methods (focus group discussions) were used in 18 countries. Quality control of data collected was undertaken by external monitors.

**Results:**

The applicability of the DF/DHF/DSS classification was limited, even when strict DHF criteria were not applied (13.7% of dengue cases could not be classified using the DF/DHF/DSS classification by experienced reviewers, compared to only 1.6% with the revised classification). The fact that some severe dengue cases could not be classified in the DF/DHF/DSS system was of particular concern. Both acceptance and perceived user-friendliness of the revised system were high, particularly in relation to triage and case management. The applicability of the revised classification to retrospective data sets (of importance for dengue surveillance) was also favourable. However, the need for training, dissemination and further research on the warning signs was highlighted.

**Conclusions:**

The revised dengue classification has a high potential for facilitating dengue case management and surveillance.

## Background

Dengue is the most rapidly advancing vector-borne disease, with an estimated 50 million infections occurring annually [1]. Its geographical spread has increased over the years to now include most countries in the tropical belt. The disease has shifted from a predominantly paediatric disease to a disease affecting all age groups. The understanding of dengue's hallmark pathophysiology has also changed; it is now recognized as plasma leakage-related rather than haemorrhage-related. Thus, the existing terminology - with its focus on haemorrhage - can be misleading for clinical management.

As dengue spreads worldwide, it has become evident that the classification of the disease into DF, DHF (Grades 1 and 2), and DSS (DHF Grades 3 and 4) [2] may not be universally applicable for clinical management [3-7]. In 2006, the WHO Dengue Scientific Working Group [8] recommended strengthening research on optimizing clinical management, in particular on the development and validation of dengue diagnostics and the analysis of new methods and guidelines for triage and care of dengue patients.

A multi-centre study including a comparative analysis of dengue clinical guidelines (DCG) from 13 countries was carried out in 2009 to evaluate the variation in use of the current DCGs in both Latin America and Asia [9]. The differences observed in the use of DCGs across countries revealed in that study suggested a need to re-evaluate and standardize dengue clinical guidelines, particularly dengue case classification and case management. Within this context, data from a prospective clinical dengue study (DENCO) in 7 countries of South Asia and Latin America served to optimize case detection and case classification into non-severe and severe categories.

**Figure 1:**
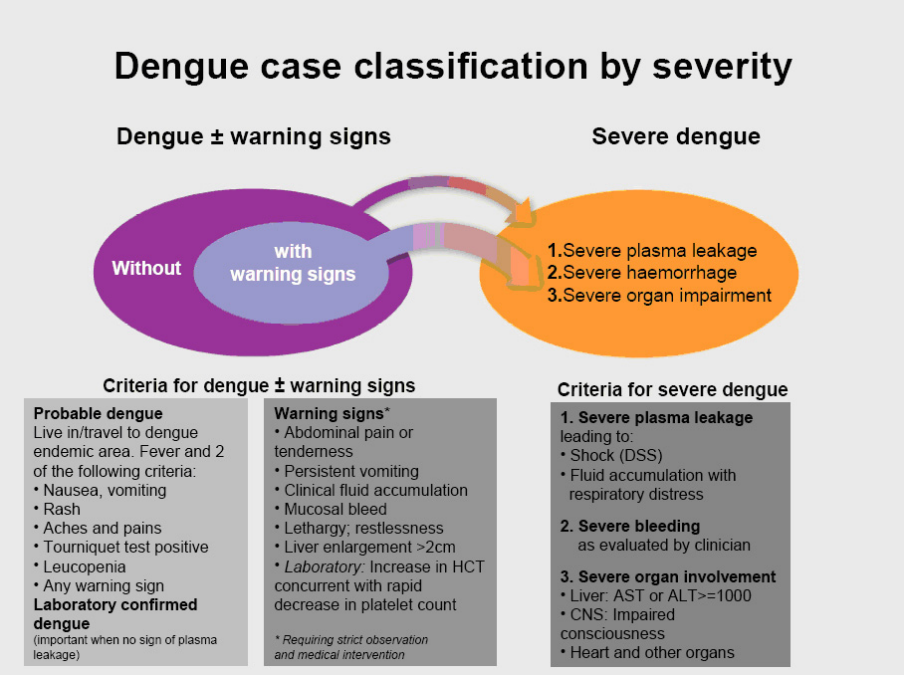
**The revised dengue case classification**. (Source: Dengue Guidelines for diagnosis, treatment, prevention and control, New edn. Geneva: WHO; 2009)

A series of regional and global expert meetings was organized by the WHO-based Special Programme for Research and Training (TDR) recognizing the evidence for a classification of dengue disease into levels of clinical severity [10], which was largely reconfirmed by the findings of a later prospective-retrospective analysis [11]. The global expert meeting on dengue classification in September 2008 in Geneva recommended a revised case classification into dengue and dengue with warning signs, and severe dengue (figure 1). This recommendation was based on (a) research evidence (from the DENCO study [10]) that showed a clear distinction between severe dengue and dengue criteria, as well as on (b) expert opinion distinguishing between dengue with and without warning signs. The definition of "dengue without warning signs" was largely based on the dengue case definition of the previous WHO dengue guidelines from 1997 [2]. The expert meeting recommended further research related to (a) the applicability and usefulness of the revised dengue classification (the subject of the current paper) in comparison with the DF/DHF Grades 1 and 2/DSS Grades 3 and 4 classification, (b) the predictive value of warning signs for patients who progressed to severe dengue (a multi-centre study to be started in 2010) and (c) an improved evidence-based definition of "probable dengue" (to be included in the predictive value study).

The study on usefulness and applicability of the revised classification presented in this paper had the following objectives:

1. To analyse the revised dengue classification in comparison to the DF/DHF 1 and 2/DSS 3 and 4 classification in relation to: (a) applicability in clinical practice, (b) usefulness for triage and clinical management, (c) user-friendliness and acceptance by health staff.

2. To analyse the applicability of the revised classification to retrospective data sets.

## Methods

### Study design and timelines

The study design, protocol and data collection instruments were developed jointly through a communication blog among 18 countries from four WHO regions (Eastern Mediterranean Region (EMR), American Region (AMR), South-East Asia Region (SEAR) and Western Pacific Region (WPR). The participating countries were located either in Latin America (12 countries) or Asia (5 countries plus one Eastern Mediterranean country). Countries were selected in a competitive selection process by an independent scientific committee. Unfortunately two countries in the Mekong delta could finally not take part due to administrative or financial reasons. Tertiary and secondary hospitals, as well as health centres at primary care level, were included at each study site.

The study was implemented from February to November 2009. A mixed methodology of quantitative and qualitative data collection was used (see below).

#### Intervention method

##### Treatment algorithm and training

A case management algorithm was developed and pilot tested in the Philippines. The case management algorithm guides the physician/nurse on what do in terms of diagnosis, treatment and patient monitoring for each patient group (A, dengue without warning signs; B, dengue with warning signs; and C, severe dengue) [1]. A standardized training package consisting of two PowerPoint slide series about diagnosis and case management according to levels of severity was developed and agreed upon.

The revised dengue classification (figure 1) and the case management algorithm were presented to dengue physicians and nurses dealing with dengue patients in each study site [primary health care (PHC) centres and hospitals] during a day of standardised training at the beginning of the study; the two series of PowerPoint slides were used during this training. Physicians/nurses/other healthcare personnel were also provided with wall posters and flyers for their daily use.

##### Data collection and research methods

**Figure 2:**
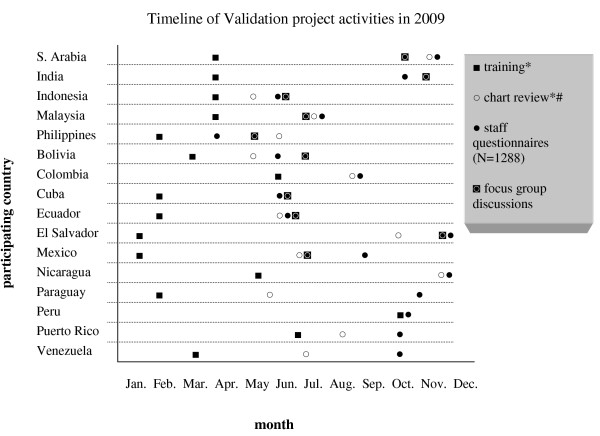
**Timeline of training and completion of the different data collection instruments in the year 2009**. • as by last day of respective activity. # only sites with prospective data

Three to six months after the initial training, health care personnel were interviewed, while their use of the DF/DHF 1 and 2/DSS 3 and 4 and of the revised severity classification was assessed through the analysis (by an experienced clinician) of treatment charts (see below). In some sites (with no or little inter-epidemic transmission of dengue), only retrospective analysis was performed (by applying the case classification to existing data sets [2^nd ^objective]). Figure 2 provides a list of the participating countries and the timeframe of project activities.

The following data collection tools were used: (1) medical chart reviews (prospective and/or retrospective), (2) self-applied questionnaires for health care personnel and (3) focus group discussions (FGD) with health care personnel.

##### Medical chart review

Charts from previous years were reviewed retrospectively while those that were started in 2009 (after the training was carried out in the institution) were reviewed prospectively. Reviewers were medical experts familiar with both the old and the revised case classification. These experts were selected on the basis of international recognition for their clinical expertise in dengue management. One reviewer was responsible for Latin America and three for South-East Asia.

Prospective reviews were carried out after individual patients had been discharged. Data was collected using a detailed case report form (CRF) which included sections on demographic information, clinical signs and symptoms, treatment, laboratory confirmation and outcome. Participating clinicians followed their local standards for the laboratory diagnosis of dengue; in some cases the proportion of laboratory-confirmed dengue was high and in others it was low (see discussion).

##### Self-applied staff questionnaires

The staff questionnaire focused on the main issues surrounding the use of current dengue guidelines (as shown in a recent analysis of dengue guidelines [9]) and the recent experience of staff when applying the revised dengue classification and case management algorithm (compared to the DF/DHF/DSS classification). Questionnaires in English or Spanish (depending on the region) were distributed to 50 healthcare staff per facility (PHC units and/or hospitals) from each site. The sample size was calculated to be a minimum of 43 health care staff per facility from each site, based on the hypothesis that at least 80% of staff (with a precision of +/- 12% at a 95% confidence interval) would report positive experiences with the revised classification. The questionnaires were filled by the staff members themselves and handed back on the same day to maximize participation.

##### Focus Group Discussions with health staff

Sample individuals for focus group participation were selected from two groups: (1) staff from health care facilities (from primary to tertiary level, but not limited to the public sector) in all study sites and (2) all available health personnel dealing with dengue treatment (e.g. health care workers, nurses, doctors, communicable disease control staff, epidemiologists and management staff). A facilitator and an assistant visited each study location according to scheduled appointments in order to conduct the FGDs. Participants were reassured about the confidentiality of any information they would give and were asked to sign a consent form. No financial incentives were given to the participants. The goal of the FGD was explained: to assess staff experience and level of comfort regarding the use of the revised case classification system, the clinical management algorithm and clinical guidelines compared to the DF/DHF 1 and 2/DSS 3 and 4 classification. Each discussion (duration approximately 1-2 hours) was conducted in the local language and covered a list of key topics. The discussions were recorded using a standard cassette recorder and information was transcribed at each individual site. The information from all sites [12] was coded and classified according to categories that emerged from the data [13]. The information was collapsed to allow for a summary, and care was taken to disregard deviant statements [14].

### Data management and analysis

#### Quality control

Data monitoring was carried out in the study sites once or twice during the study period by specially trained external monitors who checked for correct application of the study protocol and correct transfer of information from the medical charts to the CRF. This ensured comparability of the data collected from each study site and ensured that good clinical practices (according to standards) were applied.

#### Data management and analysis

Data were entered into the computer using a common data entry programme EpiInfo. Data cleaning was first done at country level. Data were then sent to the data management centre at the University Hospital Heidelberg in Germany, where the data sets were merged into one database which was then analyzed using STATA 9.2 (STATA Corporation, Duxbury, CA).

The focus group results were taped, transcribed and analysed according to topic areas (categorical analysis). Preliminary results were shared with participating countries in two regional meetings and public health messages were discussed. The final results were shared and agreed by all the principal investigators from all of the study sites.

### Ethical clearance

Ethical approval was obtained from the WHO Ethical Review Committee as well as from the local institutional review boards at each study site. As mentioned, informed consent was obtained from all study participants and information obtained from the subjects was treated as confidential.

## Results

### Chart reviews

A total of 3248 medical chart reviews were received and analysed from the 18 study sites. In Asia, 13% of the charts were collected at PHC level, 33% at secondary level and 54% at tertiary care level (in Singapore most charts were collected at PHC level while in Indonesia and Saudi Arabia most charts were collected at the secondary level). In Latin America, 30% of charts were collected from primary level, 18% from secondary level and 49% from tertiary level (3% were missing or not determined). Chart reviews were carried out both prospectively and retrospectively. Retrospective chart reviews were applied to patient charts obtained from the time period before the training course for the revised classification/case management was initiated (N = 1156), and prospective chart reviews were applied to charts which were collected after the training course and analysed at the end of the study period (N = 2092). The details are presented in table 1. The following points are worth being highlighted:

**Table 1:** Number of staff questionnaires (Q), focus group discussions (FGD) and chart reviews by country/hospital site

Country	N Staff Q	N FGDs	Chart Reviews			
			N (m/f*)	Prospective review (% of all charts)**	Mean age (SD)	laboratory-confirmed
India	54	7	148 (106/42)	0	29 (14.4)	146 (99%)

Indonesia	115	6	389 (207/182)	303 (78%)	16 (10.8)	98 (25%)

Malaysia	92	5	353 (233/120)	336 (95%)	26 (14.4)	234 (66%)

Philippines	101	18	347 (174/173)	337 (97%)	14 (9.0)	0 (0)

Singapore	0	0	103 (60/43)	0	40 (14.2)	103(100%)

Saudi Arabia	187	6	299 (164/35)	179 (90%)	31 (13.0)	174 (87%)

**Asia subtotal**	**549**	**42**	**1539 (944/959)**	**1155 (75%)**	**23 (14.6)**	**755 (49%)**

Bolivia	137	5	256 (115/141)	194 (76%)	23 (15.3)	142 (55)

Brazil	0	0	94 (46/48)	0	37 (18.6)	93 (99)

Colombia	21	0	141 (68/73)	76 (54%)	14 (16.0)	59 (42)

Cuba	38	3	100 (39/61)	0	26 (19.3)	98 (98)

El Salvador	90	3	60 (30/30)	60 (100%)	8 (6.1)	56 (93)

Ecuador	70	4	72 (42/30)	54 (75%)	24 (18.9)	60 (83)

Mexico	58	0	38 (22/16)	38 (100%)	34 (19.2)	21 (55)

Nicaragua	108	2	528 (267/261)	346 (66%)	9 (6.9)	475 (90)

Paraguay	111	3	92 (36/56)	13 (14%)	36 (19.2)	46 (50)

Peru	80	0	117 (63/54)	39 (33%)	26 (16.4)	21 (18)

Puerto Rico	9	4	58 (30/28)	42 (72%)	27 (19.1)	31 (53)

Venezuela	17	5	153 (75/78)	75 (49%)	12 (11.3)	12 (8)

**Latin America subtotal**	**739**	**29**	**1709 (833/876)**	**937 (55%)**	**19 (17.0)**	**1114 (65%)**

**Grand Total**	**1288**	**71**	**3248 (1777/1471)**	**2092 (64%)**	**21 (16.0)**	**1869 (58%)**

* m/f: male/female ratio; ** all charts include retrospective and prospective reviews

• Most countries performed both prospective and retrospective chart reviews, and included staff interviews as well as FGDs.

• Three countries (North India, Singapore, Cuba) where dengue shows a predominantly epidemic pattern with little transmission in the inter-epidemic periods applied the analysis exclusively to retrospective data sets. One country, Brazil, which was particularly interested in the use of the revised classification in dengue surveillance, did the same. Two countries (Singapore and Brazil) focused on the retrospective analysis of existing data sets only and did not include staff interviews.

• The mean age of dengue patients in the chart reviews was dependent on the type of hospital/health centre (paediatric or general).

• The laboratory confirmation of dengue was 58% on average, being higher in Latin America (65%) than in Asia (49%). Cases were considered laboratory-confirmed if positive either by PCR, by paired IgM, paired IgG (acute and reconvalescent sera) or single IgM tests.

#### Findings in the chart reviews

##### Completeness of information from routine practice to classify dengue

In order to analyse the applicability of the "dengue with warning signs" category of the revised case classification, the completeness of warning signs written in routine medical charts (prospective and retrospective assessments) were checked. These included: abdominal pain or tenderness; persistent vomiting; clinical fluid accumulation (e.g. clinical pleural effusion or ascites); bleeding from mucosal surfaces; lethargy/restlessness; and liver enlargement. In view of the DF/DHF 1 and 2/DSS 3 and 4 classification, it was evaluated whether peak and baseline haematocrit and minimal platelet counts - which are essential for diagnosing DHF - were available from routine medical charts. It could be shown that:

• Where a prospective analysis was carried out, potential warning signs were mentioned as being present or absent in the majority of charts (93.1%; 1948/2092).

• In countries with mainly or exclusively retrospective analysis consistent information on warning signs was not found in the charts.

• The tourniquet test - an important element in the early diagnosis of DHF - was carried out in only 44.9% of the prospective patients (939/2092). This was more frequent in Latin America (64.0%; 600/937) than in Asia (29.3%; 339/1155).

• Both retrospective and prospective analysis revealed that essential information for establishing the DHF diagnosis was present only in a subset of medical charts:

○ Tourniquet test: 55% (54% in retrospective; 55% in prospective)

○ Hematocrit (maximum): 11% (9% in retrospective; 12% in prospective)

○ Hematocrit (baseline): 53% (54% in retrospective, 52% in prospective)

○ Fluid accumulation: 30% (35% in retrospective; 27% in prospective)

○ Thrombocytopenia: 6% (6% in retrospective; 6% in prospective)

##### Applicability of the DF/DHF/DSS and revised classification to prospective data sets

As mentioned earlier, all medical charts completed by the treating physician were re-evaluated by an expert reviewer. In this analysis only the 2092 charts with prospective data were included. Four hundred and sixty-two of these patients referred to in these charts had manifestations of bleeding (22.1%; 462/2092); and 7.7% (162/2092) had shock according to the clinical definition. After excluding charts with missing values, 1962 of the patient charts were included in the following analysis (Table 2).

**Table 2:** Comparison of the current (DF/DHF/DSS) and the revised classification in 1962 prospective chart reviews (130 charts with missing information excluded)

DF/DHF/DSS classification by expert reviewer	Revised classification by expert reviewer				Total
	Not classifiable	Dengue		Severe Dengue	
		WS negative	WS positive		
Not classifiable	23 (8.6%)	57 (21.3%)	159 (59.3%)	29 (10.8%)	268 (100%) (13.7% of all)

DF	7 (0.5%)	551 (41.8%)	684 (51.9%)	75 (5.7%)	1317 (100%) (67.1% of all))

DHF (grades 1 and 2)	2 (0.7%)	8 (2.8%)	240 (83.0%)	39 (13.5%)	289 (100%) (14.7% of all)

DSS (DHF grades 3 and 4)	0 (0%)	0 (0%)	12 (13.6%)	76 (86.4%)	88 (100%) (4.5% of all)

Total	32 (1.6%)	616 (31.4%)	1095 (55.8%)	219 (11.2%)	1962 (100%)

* not classifiable = classification was not possible

In terms of applicability, the revised classification captured more cases than the DF/DHF 1 and 2/DSS 3 and 4 classification, as 13.7% (268/1962) of dengue patients could not be classified in the DF/DHF/DSS system because of missing information while only 1.6% (32/1962) of patients could not be classified in the revised system.

A cross-tabulation (see table 2) shows how the categories of the DF, DHF grade 1 and 2, DSS grade 3 and 4 classification match the categories of the revised classification (dengue and severe dengue). According to the judgment of the expert reviewer, DF cases were spread over different severity categories in the revised classification: 5.7% of DF cases were actually classified as "severe dengue" and 93.7% of DF cases were classified as "dengue" (with 41.8% of DF cases in the "dengue -WS" category and 51.9% in the "dengue +WS" category). A high proportion of DHF cases were also classified as "dengue" by the expert reviewer, 83% of which fell in the "dengue +WS" category. Most DSS cases (86.4%) were classified in the "severe dengue" category; however 12 DSS cases were found in the "dengue" group (all of them in the "dengue +WS"). Detailed analysis showed that only one of these 12 DSS cases had actual shock, but that all 12 had warning signs (as listed in the revised classification) and hence were correctly classified as "dengue with warning signs". Patients with shock (according to the clinical definition of pulse pressure below 20 mm Hg and/or hypotensive for age) account for 7.7% (N = 162/2092) of the total in the prospective chart reviews. Only 60 of these (37.0%) were classified as DSS but 145 (89.5%) were classified as "severe dengue", underlining the difficulty of applying the DSS definition (which is linked to DHF criteria). Thrombocytopenia was present and documented in 106 cases (72%), and bleeding tendency/tourniquet test was positive in 97 cases (60%) of patients with shock. Eleven patients (7%) did not exhibit either thrombocytopenia or bleeding tendency.

A high percentage of the severe dengue cases according to the revised classification were lab confirmed (183 of 219; 70.3%) - including those classified as DF by the expert reviewer (71 of 75). The majority of these cases was treated in a tertiary hospital (64 of 75) or came from Nicaragua (65 out of 75).

Out of the 684 patients classified as DF by the current classification and "dengue +WS" by the revised classification (see table 2), 54.4% were laboratory confirmed, 61.7% came from Asia, and 58.5% were treated at a tertiary care facility.

Out of the 8 patients classified as DHF by the current classification and "dengue - WS" by the revised classification (see table 2), 7 were lab confirmed; they are distributed over different countries - mostly in Latin America - and were treated in secondary as well as tertiary care facilities (4/4).

##### Applicability of the DF/DHF/DSS and revised classification to retrospective data sets

Eleven thousand and fifty-six charts were included in the retrospective analysis, of which 850 had complete information. The analysis showed similar results to the prospective assessment:

• A substantial proportion of cases (12.5%; 106/850) could not be classified with the DF/DHF/DSS system and a small proportion (3.1%; 26/850) could not be categorized with the revised classification system. A high proportion of non-classifiable cases with DF/DHF/DSS belonged to the category "severe dengue" under the revised classification system (32.1%; 34/106).

• DF and DHF were spread over the 3 severity levels of the revised system (examples: 81.9% of 116 DHF cases were "dengue +WS" and the reminder in the other two categories; 47.1% of 610 DF cases were under "dengue -WS" and 47.5% under "dengue +WS").

• As in the prospective analysis, a small proportion of DSS cases (16.7%; 3/18) was not classified as "severe dengue" but as "dengue +WS" and in fact did not fulfil the shock criteria.

### Staff opinion about the DF/DHF/DSS, revised case classification and treatment algorithm expressed in written interviews

#### Characteristics of respondents and availability of dengue guidelines

One thousand two-hundred eighty-eight staff questionnaires were obtained in 16 study countries; 549 in Asia and 739 in Latin America. Most respondents were clinicians (74.1% of all respondents; N = 954) or nurses (21.8% of all respondents; N = 281). The proportion of nurses was similar at primary, secondary and tertiary level, but the proportion of clinicians was particularly high at tertiary level (80.6% of all respondents at tertiary level; N = 602).

Of all respondents, 90.8%; N = 1169) had experience in dengue care in their current position (91.8% in Asia and 90.1% in Latin America). Most respondents had received some kind of clinical training in dengue (on average 63.1%; 48.6% in Asia and 73.9% in Latin America). Forty-two percent of all respondents had been working with dengue patients for 1 to 5 years, 27% for more than 5 years, and 23.7% for less than one year. More than half of all the respondents (55.4%) had seen less than 50 patients in the previous year, while 24.7% had seen more than 50 patients and 7.7% had seen none.

The most common source of dengue clinical guidelines were Ministries of Health (68.4%), followed by WHO Regional Offices (59.9%) and health facilities themselves (30%).

#### Actual use of the two classification systems

Sixty-five percent of respondents (843/1288) stated that they had used the revised classification. Of these, 83.3% (95% CI: 80.6-85.7) found the current guidelines with the DF/DHF/DSS system useful and 96.1% (95% CI: 94.5-97.3) found the revised classification useful. In Asia, 90.4% (95% CI: 87.1-93.1) of respondents were satisfied with the DF/DHF/DSS system and 96.6% (95% CI: 94.3-98.1) with the revised classification. In Latin America, 76.7% (95% CI: 72.4-80.5) of respondents found the DF/DHF/DSS system useful and 95.7% (95% CI: 93.3-97.4) considered the revised classification to be useful. Differences of opinion on the two classification systems were statistically significant.

The following sections about staff opinions regarding the revised classification and treatment algorithm show many similarities with the results obtained by the FGDs. In order to avoid repetition, and to facilitate the overview of answers given, a table with examples of answers from the staff questionnaires and FGDs is available under additional file 1.

#### Positive and negative opinions regarding the revised case classification

Table 3 provides a summary of staff opinions regarding the perceived advantages and disadvantages of the revised case classification; 75.9% are positive while 24.1% had negative comments.

**Table 3:** Perceived advantages and disadvantages regarding the revised dengue case classification (N = 1413 comments in 1288 staff questionnaires)

Advantages of the revised classification	N (%)
It helps improving management and treatment	319 (22.6%)
More simple and practical	199 (14.0%)
Easier to classify according to severity	176 (12.6%)
Easier to understand	71 (5.0%)
It helps improving triage and referral	45 (3.2%)
No disadvantages of the revised classification	191 (13.5%)
Other advantages	72 (5.0%)

*Total of positive responses*	*1073(75.9%)*

**Disadvantages of the revised classification**	**N (%)**

No advantages of the revised classification	25 (1.8%)
Needs more training and dissemination	67 (4.7%)
It's less specific. Needs more clinical entities and concise protocols	54 (3.8%)
Lack of manpower and resources	45 (3.2%)
Over diagnosis of dengue (saturation of hospitals)	32 (2.3%)
Warning signs: Too many, subjective, also in other diseases	24 (1.7%)
Lack of laboratory support	10 (0.7%)
Other disadvantages	83 (5.9%)

*Total of negative responses*	*340(24.1%)*

When specifically asked for negative experiences with the revised classification, only 20 respondents (4% of all) responded with specific comments, mainly pertaining to the wide range of warning signs and symptoms, symptoms being vague and also associated with other diseases, and the absence of shock syndrome not alerting the treating doctor.

When asked if the revised classification is useful for the classification and triage of patients compared to the DF/DHF/DSS classification, the most frequent answers were that (a) it simplifies case management and leads to adequate treatment (N = 162), (b) it allows for a more precise dengue classification including severity (N = 97), and (c) it is simpler, more practical and user friendlier (N = 91).

#### Staff opinions regarding the treatment algorithm based on the revised case classification

Six hundred and ninety-eight comments were received regarding the applicability of the treatment algorithm; 83% were positive about the algorithm. However, the remaining responses (17%) referred to the lack of staff to apply the guidelines, poor training, difficulty in accessing the documents (in Latin America only), and unavailability of diagnostic tests.

When asked for recommendations regarding the extended use of the treatment algorithm across countries and continents, there was a feeling that more training and dissemination of the revised classification and treatment algorithm were needed (53.4% out of 698 responses). Respondents also suggested better access to diagnostic tests (20.2%), better training (38.7%) and the need for more concise parameters for treatment interventions (18.2%).

### Staff opinion about the DF/DHF/DSS classification, revised case classification and treatment algorithm expressed in Focus Group Discussions

Thirteen countries held 71 FGD in total. Personnel from 75 health care facilities attended. The participants in the focus groups - mostly medical staff and nurses involved in dengue care, but also in some cases epidemiologists and public health staff - had received training about the revised dengue case classification and treatment algorithm 3 to 6 months before the FGD. Observer bias was reduced by tape recording of the interviews with subsequent transcription and categorical analysis (see methods).

Staff perceptions about (a) positive and (b) negative aspects of the revised classification as well as about (c) applicability of the treatment algorithm (based on the revised classification) and staff recommendations will be presented:

#### a) Perceived usefulness of the revised case classification

Across all study sites, the revised case classification received positive comments, particularly regarding its ease of use and focus on clinical management: *"The revised case classification is very practically oriented, didactic, has a good and clear scheme, is easy and clear" *(Bolivia). Advantages were frequently mentioned: *"The term hemorrhagic has been generally misunderstood" *(Philippines), the *"...epidemiological profile can now be better described" *(Cuba/Ecuador), *"The classification reflects the dynamic changes of the disease" *(India), there is *"...a reduced need for laboratory testing" *(Paraguay), and the *"...incorporation of warning signs..." *is perceived to be useful for clinical management (El Salvador).

Comments from epidemiologists involved in the FGD were equally positive: "For epidemiologists and surveillance officers, the revised guidelines will be able [sic] to establish consistency of data entries for both hospital statistics and morbidity/mortality rates" (Philippines).

In all sites, there was a general view that the revised case classification is better for case management and triage compared to the DF/DHF/DSS system.

#### b) Perceived limitations of the revised classification

Some concerns were raised about the use of the revised classification system. These were site specific and not as general as the positive comments:

• One of the difficulties will be the need for staff training in the introduction phase of the revised classification.

• *"There may be a need for local adaptation of some elements" *(Cuba/Ecuador).

• *"23 out of 40 participants would not consider the increasing haematocrit with decreasing platelet count as a warning sign, but rather it should be defervescence" *(Philippines).

• *"Initially we thought to have more admissions but this was not the case" *(Malaysia).

• Epidemiological data collection needs to be updated and the surveillance system needs to be adapted.

#### c) Applicability of the treatment algorithm

In most countries, no algorithm for triage and dengue case management existed prior to this study. Therefore, most medical staff welcomed the decision-making algorithm as practical support. Positive comments (across all sites in Latin America and Asia) highlighted the advantages of having clear triage criteria (especially with warning signs), the reduced requirement of laboratory testing, the compatibility with the concept of Integrated Management of Childhood Illness and the recognition of social factors. For example in the Philippines: *"All of the 40 participants said that the algorithm is simple and easy to follow and is very useful in triaging patients, classifying dengue and therefore managing the disease"*, or in Puerto Rico where immediate action was taken: *"Previously existing guidelines are already updated to include the revised case classification"*, or in Colombia: *"The advantage I can see is the clear link between classification and case management"*.

Some concerns were related to the following issues:

• The introduction of change is always difficult and will require extra efforts in the implementation.

• In countries with existing treatment flowcharts (algorithms) including cut-off level of 100,000 platelets, this was also perceived to be useful for decision-making.

• Harmonization between different existing guidelines is needed, since there are different WHO, regional and national guidelines circulating (The Philippines).

• In high endemicity countries, the unrestricted use of warning signs may lead to unnecessary admissions (Indonesia).

## Discussion

The present study used a methodological mix of quantitative, semi-quantitative and qualitative methods to determine the applicability of the revised dengue case classification system in 18 countries. Efforts were made to reduce observer bias and ensure robustness of data as discussed below.

### Applicability of a dengue classification system in the clinical practice (objective 1)

The limited applicability of the DF/DHF Grade 1 and 2/DSS Grades 3 and 4 classification in clinical practice (particularly at PHC level), due to the rigidity of classification criteria and dependence on laboratory tests, has been highlighted in literature reviews [6]. The prospective clinical DENCO study [10] further showed that in ~18% of dengue cases a correct application of the DHF criteria (fever, hemorrhagic tendencies, thrombocytopenia and plasma leakage expressed as raising haematocrit, pleural effusion and other signs and symptoms) was not possible. This has apparently lead to the clinical practice of diagnosing DHF without all the necessary criteria and the application of locally adapted variations [9]. Our study highlights these issues. A large proportion - usually above 50% - of DHF criteria (such as haematocrit before and after treatment, platelet counts, and tourniquet tests), were not collected by the clinician; however, the expert reviewer of the medical charts used clinical judgement to come to the DHF diagnosis. But even doing so, 13.7% of patients could not be classified into the DF/DHF/DSS categories.

For the revised classification only 1.6% of dengue cases could not be classified and the warnings signs for severe disease (necessary for the "dengue +WS" category) were documented in a large proportion of medical charts (see the section on "completeness of information" in the results).

The revised classification also proved to be more sensitive for timely recognition of severe disease. Prospective chart reviews across all three levels of the health care system showed that a significant proportion of cases classified as DF by the DF/DHF 1 and 2/DSS 3 and 4 classification are picked up as potentially severe or severe (dengue plus warning signs or severe dengue) by the revised classification. In a direct comparison of the two classification systems, 51.9% (684/1327) of the DF cases were classified as dengue plus warning signs (revised classification) and another 5.7% (75/1327) as severe dengue (revised classification). Mismatches in the other direction, where cases were actually classified more severe by the DF/DHF 1 and 2/DSS 3 and 4 compared to the revised classification, were only present in a very small proportion, most of them being cases of DHF that were classified as "dengue without warning signs" according to the revised classification (N = 8, according to expert reviewer).

### Applicability of a dengue classification system in dengue surveillance (retrospective chart review (objective 2)

The difficulties of applying the DF/DHF/DSS system in dengue surveillance are documented [6]. In this study, the applicability of the two classification systems to retrospective data sets was tested. Here again the proportion of "non-classifiable" cases was higher when applying the DF/DHF/DSS system (12.5%) than when applying the revised case classification (only 3.1%). It is of particular concern that 32.1% of severe dengue cases could not be classified in the DF/DHF/DSS system. The completeness of information, particularly on potential warning signs, was lower in the retrospective chart review than in the prospective one - contributing to the 3.1% which could not be classified.

### Usefulness of a severity classification for triage and case management (objective 1)

During an outbreak simple criteria for triage and case management are required to decide if patients can be treated at home, in a hospital ward or require intensive care. During inter-epidemic periods, endemic or sporadic transmission remains and triaging dengue cases for case management remains important for organizing health services. Our study is in line with the findings of the prospective DENCO study that DF, DHF and DSS only match to a limited degree with a more clinically oriented classification of severity. The revised classification was easily applicable in clinical practice (see above) but was also seen to be useful for triage and case management by medical staff, more frequently than the DF/DHF/DSS classification.

Yet, in the focus groups and questionnaires, some concerns on the revised classification were raised, these included:

• hospitalization rates might increase if the warning signs are not precisely defined and therefore the need for a prospective study on the definition and usefulness of warning signs across different countries and health care levels was emphasized

• cost implications if more patients are being admitted

• the need for more training and dissemination and for more concise clinical protocols

The question if the revised classification leads to higher patient numbers cannot be answered with the results from this study and remains to be clarified. Issues related to triage and case management will have to be addressed in future studies.

### Potential bias/limitations

The term prospective vs. retrospective refers to the timing of the training versus the treatment of the patients by the treating physician. Reviewers' assessment of the patient's classification was based on the medical chart after discharge of the patient. Thus, the knowledge of the outcome could theoretically have influenced the reviewer's assessment. However, as the reviewer had to check the presence of one or more items to classify severe dengue (revised classification) or a set of four items for DHF (current classification) the potential bias would most probably have worked in favour of the current classification in the sense that cases with severe clinical course would receive special attention to investigate the presence of all items needed to fulfil DHF in case of the old classification.

### Acceptance and user-friendliness of a dengue classification system (objective 1)

Staff interviews and FGDs suggested that the level of acceptance for the revised classification was high. This was particularly the case for Latin America. Surprisingly it was also high in Asia, where the DF/DHF/DSS classification has a long tradition [15,16].

The staff questionnaires also revealed that the revised classification was perceived as being more user-friendly compared to the DF/DHF/DSS classification, and that this has a direct implication on adequate case management and treatment. The respondents appreciated the revised classification with its emphasis on clinical severity which led directly to specific case management instructions in the clinical management algorithm. There was also an agreement across countries and through all methods used that the use of warning signs for case management, as reflected in the revised classification, is of practical value. However, it is obvious that both the definition of "warning signs for severe dengue" and the predictive value of these signs require further research. In addition, the question of a (clinical) dengue case definition in the absence of confirmatory laboratory tests needs further research. Taking into account the lessons learned from development of the revised classification, the development and dissemination of detailed guidelines on dengue clinical management is crucial - with emphasis placed on training for health staff at all levels. Furthermore, as suggested in a number of staff interviews, the recognition of special situations such as dengue in pregnancy, dengue and co-morbidities, dengue in paediatric and adult care need further consideration.

While there is a need for harmonizing guidelines (see [9]), it also may well be that algorithms in the dengue case definition may vary from region to region or even from country to country, and that some warning signs for severe dengue may show geographical variation. This will require the adaptation of dengue clinical guidelines to local characteristics. However, this study has shown that the revised dengue classification is suitable for clinical practice. For dengue surveillance, there is potential for the revised classification to lead to simpler, more consistent, and comparable data on dengue and severe dengue.

## Conclusions

The revised dengue classification has a high potential for facilitating dengue case management and surveillance. It was shown and perceived to be more sensitive than the DF/DHF/DSS classification for timely recognition of severe disease. Both acceptance and perceived user-friendliness of the revised system were high, particularly in relation to triage and case management. The applicability of the revised classification to retrospective data sets was also favourable and the proportion of non-classifiable cases overall was very low.

The need for training and dissemination in general, and for further research on warning signs and on the clinical case definition of dengue in the absence of laboratory testing in particular, was highlighted.

## Competing interests

The authors declare that they have no competing interests.

## Authors' contributions

JB participated in the design and coordination of the study, and performed statistical analysis and interpretation of merged data. RG participated in the design of the study and carried out the overall data management and statistical analysis of merged data. EVB, VCR, DS, EBPS, MP, ISL, JGM, AN, AB, IA, GR, ED, KT, NAA, EEO, EVA, TTH participated in the acquisition and analysis of local data. EM contributed to the conception and design of the study, and coordinated the introduction of the study in the Latin American region. LL participated in the conception and design of the study, and in the acquisition and analysis of local data. JF contributed to the conception and design of the study. OH participated in the conception and design of the study, carried out data monitoring, focus group discussions and interpretation of its results. AK participated in the conception, design and coordination of the study. TJ participated in the conception, design, analysis and coordination of the study, and performed interpretation of merged data. All authors were involved in the drafting of the manuscript, and all have read and approved the final manuscript

## Pre-publication history

The pre-publication history for this paper can be accessed here:

http://www.biomedcentral.com/1471-2334/11/106/prepub

## Supplementary Material

Additional file 1 Overview table of opinions regarding revised dengue classification and treatment algorithm Click here for file
