# Supplementary material for: Usefulness and applicability of the revised dengue case classification by disease: multi-centre study in 18 countries
Source: BMC Infect Dis. 2011 Apr 21;11:106. doi: 10.1186/1471-2334-11-106 (PMC3098176; doi:10.1186/1471-2334-11-106)
Supplement: Additional file 1 — Overview table of opinions regarding revised dengue classification and treatment algorithm [file 1471-2334-11-106-S1.PDF]

Additional file 1: Overview table of opinions regarding revised dengue classification and treatment algorithm

**Staff questionnaires**	**Focus Group Discussions**
**Perceived positive aspects of the revised dengue classification:**	
Easily understood, even nurses in the ward could do the monitoring of warning signs" (Philippines)	"The revised case classification is very practically oriented, didactic, has a good and clear scheme, is easy and clear" (Bolivia)
"It is a combination of clinical signs and minimal laboratory tests in the diagnosis of dengue" (Paediatrician, Philippines)	"the term hemorrhagic has been generally misunderstood" (Philippines)
"Because it further subdivides the DHF group according to the degree of severity and identifies cases with this group that should be prioritized…" (Specialist, Philippines)	"epidemiological profile can now be better described" (Cuba/Ecuador)
"Quick to consult and easy to use" (Resident, Bolivia); "In takes into account other clinical aspects other than haemorrhage" (Intern, Bolivia)	"the classification reflects the dynamic changes of the disease" (India)
"It considers the warning sings as elements of severity that precede shock" (Tertiary level, Cuba)	"There is a reduced need for laboratory testing" (Paraguay)
"It follows the natural evolution of disease" (Paediatrician, Cuba)	"the incorporation of warning signs is perceived to be useful for clinical management" (El Salvador)
"The warning sign clause is useful, more relevant. Fluids more accurate" (Internist, Malaysia)	"For epidemiologists and surveillance officers, the revised guidelines will be able to establish consistency of data entries for both hospital statistics and morbidity/mortality rates" (Philippines)
"Easy to remember and practical" (Physician, Mexico)	

“It doesn’t depend on rigid laboratory parameters like platelet count, it takes into account the visceral compromise of dengue” (Intern, Colombia)	
“It includes patients that in the old classification didn’t fulfil all criteria for severity and thus were left out of statistics and surveillance” (Resident, Nicaragua);	
“It’s mainly clinical and I believe nobody gets lost in the classification” (Paediatrician, Nicaragua).	
**Perceived negative aspects of the revised dengue classification:**	
„The signs and symptoms are vague that we can also see in other diseases" (Physician, Philippines)	"There may be a need for local adaptation of some elements such as the list of warning signs" (Cuba/Ecuador)
“Too many criteria / clinical features. All patients will have warning signs”, (Internist, Malaysia)	"23 out of 40 participants would not consider the increasing haematocrit with decreasing platelet count as a warning sign, but rather it should be defervescence" (Philippines)
“Less useful, especially for dengue without warning signs because of the wide coverage” (Physician, Indonesia)	"Initially we thought to have more admissions but this was not the case" (Malaysia)
“The new classification is not clear, for example: abdominal pain. What kind? Sudden decrease of TC and Hct, how much? Unclear, too superficial, it is easier to differentiate dengue severity using DF/DHF.” (Physician, Indonesia)	Epidemiological data collection needs to be updated and the surveillance system needs to be adapted.
“With the absence of shock syndrome, the doctor doesn’t get alerted”(Paediatrician, Ecuador)	

“They try to guide about the new classification, but the medical histories do not tabulate information of this classification” (Paediatrician, Venezuela)	
“There are signs and symptoms that don’t fit in the definition of a dengue case” (Tertiary level, Peru)	
The criteria used to diagnose the patient is too broad, mainly because all patients with fever are classified as dengue and primary health care level will saturate the secondary level.” (Resident, Nicaragua).	
**Perceived positive aspects of the treatment algorithm based on the revised case classification:**	
Question not asked	“The algorithm is simple and easy to follow and is very useful in triaging patients, classifying dengue and therefore managing the disease" (all participants)
	"The advantage I can see is the clear link between classification and case management" (Colombia)
**Suggestions to improve the treatment algorithm based on the revised case classification:**	
“Clearer Criteria for immeasurable limit to make the management easier and more applicable” (Physician, Indonesia)	Question not asked
“Please add more objective points in severe dengue category like pulse pressure, takicardia, bekicardia” (Internist, India)	
“Make the guide pocket-size” (Specialist, Paraguay)	
“Give more examples of signs and symptoms present in the disease” (Nurse, Puerto Rico)	
